# Description of six new species of *Lycocerus* Gorham (Coleoptera, Cantharidae), with taxonomic note and new distribution data of some other species

**DOI:** 10.3897/zookeys.456.8465

**Published:** 2014-11-21

**Authors:** Yuxia Yang, Junyan Su, Xingke Yang

**Affiliations:** 1 College of Life Sciences, Hebei University, Baoding 071002, Hebei Province, China; 2 Key Laboratory of Zoological Systematics and Evolution, Institute of Zoology, Chinese Academy of Sciences, Beijing 100101, China

**Keywords:** Taxonomy, *Lycocerus*, new species, synonym, new records, China, Vietnam, Myanmar

## Abstract

Six new species of *Lycocerus* Gorham are described, *Lycocerus gracilicornis* **sp. n.** (China: Sichuan), *Lycocerus longihirtus* **sp. n.** (China: Yunnan), *Lycocerus sichuanus* **sp. n.** (China: Sichuan), *Lycocerus hubeiensis* **sp. n.** (China: Hubei), *Lycocerus napolovi* **sp. n.** (Vietnam: Sa Pa) and *Lycocerus quadrilineatus* **sp. n.** (Vietnam: Sa Pa), and provided with illustrations of habitus, antennae and aedeagi of male or and antennae, abdominal sternites VIII and genitalia of female. *Lycocerus rubroniger* Švihla, 2011 is synonymized with *Lycocerus obscurus* Pic, 1916. *Lycocerus hickeri* Pic, 1934 and *Lycocerus obscurus* are provided with illustrations of abdominal sternites VIII of female. Nine species are recorded from China for the first time, *Lycocerus bicoloripennis* (Pic, 1924), *Lycocerus caliginostus* Gorham, 1889, *Lycocerus jendeki* Švihla, 2005, *Lycocerus malaisei* (Wittmer, 1995), *Lycocerus obscurus*, *Lycocerus olivaceus* (Wittmer, 1995), *Lycocerus purpureus* Kazantsev, 2007, *Lycocerus ruficornis* (Wittmer, 1995) and *Lycocerus semiextensus* (Wittmer, 1995), and *Lycocerus ruficornis* is also recorded for Myanmar for the first time.

## Introduction

*Lycocerus* Gorham, 1889 *sensu lato* (Okushima 2005) is one of the largest genera of cantharid beetles, with more than 300 species widely distributed in the Oriental and eastern Palaearctic Regions (Kazantsev and Brancucci 2007). The species of *Lycocerus* in a strict sense, which are characterized by the broad antennomeres and the red, generally densely pubescent pronotum and elytra, from Indochina and adjacent regions, were revised by Kazantsev (1999); later some similar species were added by Švihla (2011) and Okushima and Yang (2013). Following these studies, six new species from China and Vietnam, which are similar to *Lycocerus hickeri* Pic, 1934, were discovered recently. The new species are described here under the names of *Lycocerus gracilicornis* sp. n., *Lycocerus longihirtus* sp. n., *Lycocerus sichuanus* sp. n., *Lycocerus hubeiensis* sp. n., *Lycocerus napolovi* sp. n. and *Lycocerus quadrilineatus* sp. n.

Additionally, *Lycocerus rubroniger* Švihla, 2011 is considered to be a junior synonym of *Lycocerus obscurus* Pic, 1916. Besides, nine species are recorded from China for the first time: *Lycocerus bicoloripennis* (Pic, 1924), *Lycocerus caliginostus* Gorham, 1889, *Lycocerus jendeki* Švihla, 2005, *Lycocerus malaisei* (Wittmer, 1995), *Lycocerus obscurus*, *Lycocerus olivaceus* (Wittmer, 1995), *Lycocerus purpureus* Kazantsev, 2007, *Lycocerus ruficornis* (Wittmer, 1995) and *Lycocerus semiextensus* (Wittmer, 1995), and *Lycocerus ruficornis* is also a first record for Myanmar.

## Material and methods

The material is preserved in the following collections, and the primary types were returned to the collections from which they were borrowed or were otherwise deposited in public museums.

CAS California Academy of Sciences, San Francisco, USA;

IZAS Institute of Zoology, Chinese Academy of Sciences, Beijing, China;

MCSNG Museo Civico di Storia Naturale “Giacomo Doria”, Genova, Italy;

MHBU Museum of Hebei University, Baoding, China;

MNHN Muséum national d’Histoire naturelle, Paris, France;

NHMB Naturhistorisches Museum Basel, Switzerland;

NMPC Narodni muzeum, Praha, Czech Republic;

ZIN Zoological Institute, Russian Academy of Sciences, St. Petersburg, Russia.

The genitalia of both sexes and abdominal sternites VIII of females were dissected and cleared in 10% KOH solution, and the female genitalia was dyed with hematoxylin. Habitus photos were taken with a Leica M205 A microscope, multiple layers were stacked using Combine ZM (Helicon Focus 5.3). Line drawings were made with the aid of camera lucida attached to a Leica MZ12.5 stereomicroscope, then edited in CorelDRAW 12 and Adobe Photoshop 8.0.1.

Complete label data are listed for type specimens, using brackets “[ ]” for our remarks and comments, [p] indicating that the following data are printed and [h] that they are handwritten. Quotation marks are used to separate data from different labels and a backslash “\” to separate data from different lines of the same label.

Body length was measured from the anterior margin of the clypeus to the elytral apex and body width across the humeral part of elytra. Morphological terminology of female genitalia follows that of Brancucci (1980). The abbreviations in the figures are as follows, ag: accessory gland; co: coxite; di: diverticulum; tg9: abdominal tergite IX; sd: spermathecal duct; sp: spermatheca; ov: median oviduct; va: vagina.

## Taxonomy

### *Lycocerus hickeri* species-group

#### Diagnosis

Antennae (Fig. 7) nearly filiform, or middle antennomeres slightly widened apically, nearly long-triangular (Figs 8–15), present with narrow longitudinal to round smooth impressions along outer margins of antennomeres IV–XI in male. Pronotum subquadrate, with lateral margins slightly to moderately diverging posteriorly. Elytra elongate, red, more or less darkened at elytral interstices, present with more or less developed elytral venation and costate, surface rugulose-lacunose, densely and coarsely punctate, matt, combined with sparse, short, decumbent reddish-brown pubescence and much longer, semierect reddish-brown pubescence and erect black pubescence. Tarsal claws variable, either simple in both sexes, or pro- and meso-outer claws with basal projections in both sexes or in female while simple in male, or pro- and meso-inner and outer claws with basal projections in female while simple in male. Female genitalia (Figs 39–41): vagina stout and extended apically as a thick duct; diverticulum and spermathecal duct arising from the end of the duct of vagina; diverticulum moderately long, thin and spiral; spermathecal duct much thicker than diverticulum; spermatheca as thick as spermathecal duct at basal portion, abruptly narrowed apically, thin and spiral at apical portion, much longer than diverticulum, provided with moderately long and thin accessory gland.

#### Distribution

China (Yunnan, Sichuan, Hubei); Vietnam (Sa Pa).

#### Remarks

This species group could be distinguished from other species of *Lycocerus* by the characteristic sculpture and pubescence of elytra. The female genitalia of the species are very similar, but each could be differentiated by the structure of aedeagus, abdominal sternite VIII of female, antennae, pronotum and tarsal claws of both sexes.

#### Key to the species of *Lycocerus hickeri* species-group

**Table:** 

1	Pro- and meso-outer tarsal claws each with a basal projection in male	**2**
–	All claws simple in male	**4**
2	Pronotum nearly as long as wide; aedeagus: dorsal fig of each paramere with inner margin nearly arcuate	***Lycocerus longihirtus* sp. n.**
–	Pronotum distinctly longer than wide; aedeagus: dorsal fig of each paramere with inner margin bisinuate	**3**
3	Aedeagus: ventral process of each paramere even and nearly straight in lateral view	***Lycocerus sichuanus* sp. n.**
–	Aedeagus: ventral process of each paramere narrowed at base and slightly bent dorsally in lateral view	***Lycocerus hubeiensis* sp. n.**
4	Pronotum nearly as long as wide, with lateral margins slightly diverging posteriorly	**5**
–	Pronotum longer than wide, with lateral margins moderately diverging posteriorly	**6**
5	Antennae with middle antennomeres widened apically; elytra not darkened at elytral interstices, elytral venation well-developed, distinctly costate	***Lycocerus hickeri* Pic, 1934**
–	Antennae nearly filiform; elytra darkened at the second elytral interstices, elytral venation slightly developed, not costate	***Lycocerus gracilicornis* sp. n.**
6	Elytra black at all elytral interstices; pronotum distinctly longer than wide; aedeagus: ventral process of each paramere normal, nearly straight in lateral view, dorsal fig with inner margin nearly straight, outer angle obtuse-angled	***Lycocerus quadrilineatus* sp. n.**
–	Elytra black at the first and second elytral interstices; pronotum slightly longer than wide; aedeagus: ventral process of each paramere flattened and twist in middle in ventral view, distinctly bent dorsally in lateral view, dorsal fig with inner margin distinctly protuberant in middle, outer angle triangular and bent ventrally	***Lycocerus napolovi* sp. n.**

#### Taxonomy

##### Lycocerus hickeri

Taxon classificationAnimaliaColeopteraCantharidae

Pic, 1934

Fig. 34

Lycocerus hickeri Pic, 1934: 46.
Athemellus hickeri : Kazantsev, 1999: 119.

###### Type specimens examined.

Lectotype ♀ (NHMB): [p] “Asia, China”, [p] “coll. Richard \ Hicker, Wien”, [h] “hickeri n. sp.”, [p] “Athemus \ hickeri (Pic) \ det. S. Kasantsev 1996”, [p] “LECTOTYPUS”, [p] “CANTHARIDAE \ CANTH00000915”.

###### Distribution.

China.

###### Remarks.

This species was described on the basis of female types, and its locality is not accurate within China. Here the abdominal sternite VIII (Fig. 34) of the female is illustrated for the first time: it is largely and roundly emarginated in middle and both sides of posterior margin, the portion between middle and lateral emarginations acute-angled at apex.

##### Lycocerus gracilicornis

Taxon classificationAnimaliaColeopteraCantharidae

Y. Yang & X. Yang sp. n.

http://zoobank.org/7EC71583-2FC5-4151-B4E7-A6B7E4BD550F

Figs 1 , 7 , 16 –18

###### Type material.

Holotype ♂ (IZAS): CHINA, Sichuan, Yajiang to 5km of Litang, 2595m, leg. Gan-Yan Yang. Paratypes: CHINA, Sichuan: 2♂♂ (IZAS): Yajiang, Hekou, Shanbeihou, 2838m, 27.V.2009, leg. Gan-Yan Yang; 1♂ (IZAS): Yajiang, 24.V.2009, leg. Feng Yuan; 1♂ (IZAS): Yajiang, Bajiaolou, 29.V.2009, leg. Zhi-Liang Wang [the above are all transliterated from Chinese labels]; 1♂ (NHMB): “CHINA, Sichuan prov., Mts W. Bamei, 3750m, 12.08.2005, leg. S. Murzin”.

###### Distribution.

China (Sichuan).

###### Description.

**Figures 1–2.**
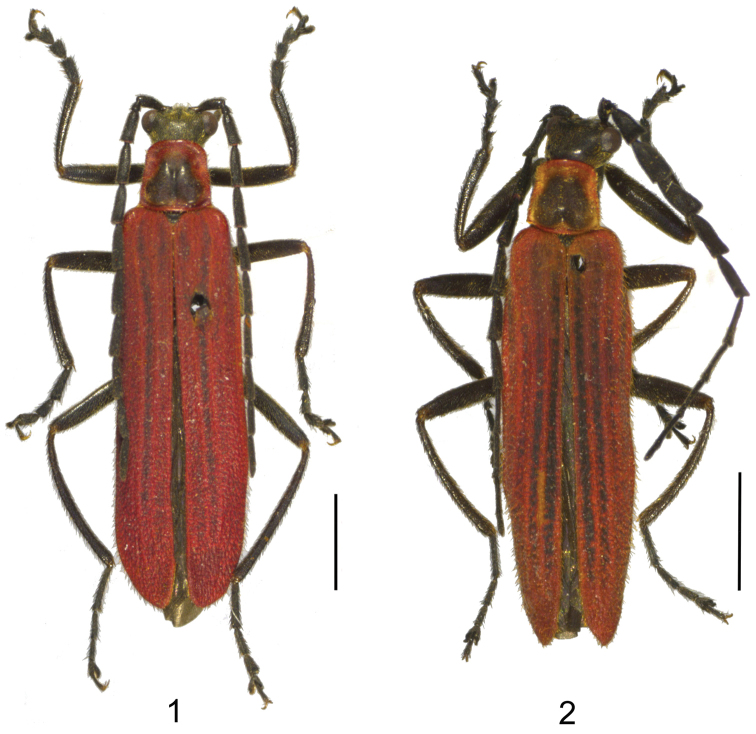
Male habitus, dorsal view: **1** *Lycocerus gracilicornis* sp. n. **2** *Lycocerus longihirtus* sp. n. Scale bars: 2.0 mm.

Male (Fig. 1). Body black, mandibles dark brown, pronotum red, with a large black marking in center of disc, elytra red, distinctly darkened almost along the whole length of the second elytral interstices, slightly darkened at the first elytral interstices.

Head evenly narrowed behind eyes, surface densely and finely punctate, semilustrous, covered with dense, fine, yellowish brown decumbent pubescence; eyes moderately protruding, head width across eyes moderately wider than anterior margin of pronotum; terminal maxillary palpomeres long-triangular, arcuate and sharp at apical one-third length of inner margins; antennae (Fig. 7) filiform, extending to apical one-third length of elytra, antennomeres II slightly longer than wide at apices, III–XI nearly parallel-sided, III about 2.5 times as long as wide, IV about one-third longer than III, XI slightly longer than X and pointed at apices, IV–XI each with a small round smooth impression at apical part of outer margin.

Pronotum nearly as long as wide, widest near posterior margin, anterior margin arcuate, anterior angles rounded, lateral margins slightly diverging posteriorly, posterior angles rectangular, posterior margin slightly arcuate and narrowly bordered, disc moderately convex at posterolateral parts, surface punctate and pubescent like that of head, semilustrous.

Elytra about 5.5 times longer than pronotum, 4.0 times as long as humeral width, which about one-third wider than posterior margin of pronotum, lateral margins nearly parallel, elytral venation slightly developed, not costate.

All tarsal claws simple.

Abdominal sternite IX long-triangular. Aedeagus (Figs 16–18): ventral process of each paramere normal and rounded at apex, distinctly narrowed at base and slightly bent dorsally in lateral view; dorsal fig slightly shorter than ventral process, abruptly narrowed at inner apical portion, with inner angle rounded, outer angle obtuse-angled, inner margin sinuate, apical margin rounded; laterophyse with apex pointed laterodorsally to outer angle of dorsal fig.

Female. Unknown.

Body length (males): 8.0–10.0 mm; width: 2.0–2.2 mm.

###### Etymology.

This specific name is derived from Latin *gracilis* (narrow) + *cornu* (horn), referring its antennomeres III–XI nearly parallel-sided.

###### Diagnosis.

This species is similar to *Lycocerus hickeri* Pic, but the antennae filiform, with antennomeres III–XI nearly parallel-sided; elytral venation less developed and the second elytral interstices darkened.

##### Lycocerus longihirtus

Taxon classificationAnimaliaColeopteraCantharidae

Y. Yang & X. Yang sp. n.

http://zoobank.org/ED7AEC94-EC7F-4D6B-9B32-39F1DD945BC6

Figs 2 , 8 , 19 –21

###### Type material.

Holotype ♂ (MHBU): CHINA, Yunnan, Yunlong, Tianchi Nat. Res., 9.VII.2011, leg. Hao-Yu Liu. Paratype: 1♂ (MHBU): same data as holotype. [Both transliterated from Chinese labels].

###### Distribution.

China (Yunnan).

###### Description.

Male (Fig. 2). Body black, mandibles dark brown, pronotum red, with a large black marking in center of disc, elytra red, black almost along the whole length of the first and second elytral interstices, more or less darkened at the third and fourth elytral interstices.

Head evenly narrowed behind eyes, surface densely and finely punctate, semilustrous, covered with dense, fine, yellowish brown decumbent pubescence; eyes moderately protruding, head width across eyes moderately wider than anterior margin of pronotum; terminal maxillary palpomeres long-triangular, arcuate and sharp at apical one-third length of inner margins; antennae (Fig. 8) extending to apical one-third length of elytra, antennomeres II nearly as long as wide at apices, III–XI flattened, III–VIII obliquely widened apically, nearly long-triangular, III about twice as long as wide at apices, IV slightly longer than III, IX–XI nearly parallel-sided, XI slightly longer than X and pointed at apices, IV–XI each with an oval to round smooth impression at apical part of outer margin.

Pronotum nearly as long as wide, widest near posterior margin, anterior margin arcuate, anterior angles rounded, lateral margins slightly diverging posteriorly, posterior angles rectangular, posterior margin slightly arcuate and narrowly bordered, disc moderately convex at posterolateral parts, surface punctate and pubescent like that of head, semilustrous.

Elytra about 5.8 times longer than pronotum, 4.0 times longer than humeral width, which about one-third wider than posterior margin of pronotum, lateral margins nearly parallel, elytral venations moderately developed, slightly costate.

Pro- and meso-outer tarsal claws each with a basal projection.

Abdominal sternite IX long-triangular. Aedeagus (Figs 19–21): ventral process of each paramere normal and rounded at apex, distinctly narrowed at base and slightly bent dorsally in lateral view; dorsal fig distinctly shorter than ventral process, evenly narrowed apically, with inner angle widely rounded, outer angle obtuse-angled, inner margin nearly arcuate, apical margin rounded, around with long pubescence; laterophyse with apex pointed laterodorsally to outer angle of dorsal fig.

Female. Unknown.

Body length (males): 8.0–9.0 mm; width: 1.8–2.0 mm.

###### Etymology.

This specific name is derived from Latin *longus* (long) and *hirtus* (hairy), referring to its aedeagus: dorsal fig of each paramere covered with long pubescence along apical margin.

###### Diagnosis.

This species is similar to *Lycocerus gracilicornis* sp. n., but the antennomeres III–VIII widened apically, nearly long-triangular; elytral venation moderately developed, slightly costate; aedeagus: dorsal fig of each paramere evenly narrowed apically, inner margin arcuate, apical margin around with long pubescence.

##### Lycocerus sichuanus

Taxon classificationAnimaliaColeopteraCantharidae

Y. Yang & X. Yang sp. n.

http://zoobank.org/36C64C4C-983D-40D0-A440-D76A11086E74

Figs 3 , 9 –10 , 22 –24 , 35 , 39

###### Type material.

Holotype ♂ (IZAS): CHINA, Sichuan, Mt. Emei, Xixiangchi, 1800–2000m, 12.VII.1957, leg. Zong-Yuan Wang. Paratypes: CHINA, Sichuan: 1♂ (IZAS): Mt. Emei, Jiulaodong, 1800–1900m, 26.VII.1957, leg. Ke-Ren Huang; 1♀ (IZAS): same locality, 31.VII.1957, leg. You-Cai Yu; 1♀ (IZAS): same locality, 30.VII. 1957, leg. You-Cai Yu; 1♀ (IZAS): same locality, 14.VIII.1957, leg. Zong-Yuan Wang [the above are all transliterated from Chinese labels]; 1♂ (NHMB): “CHINA, Sichuan prov., 70km West Chengdu, Qingcheng Hou Shan mts., 30°44'N, 103°08'E, 1500m, 8.–14.VI.2005, leg. S. Murzin”.

###### Distribution.

China (Sichuan).

###### Description.

**Figures 3–4.**
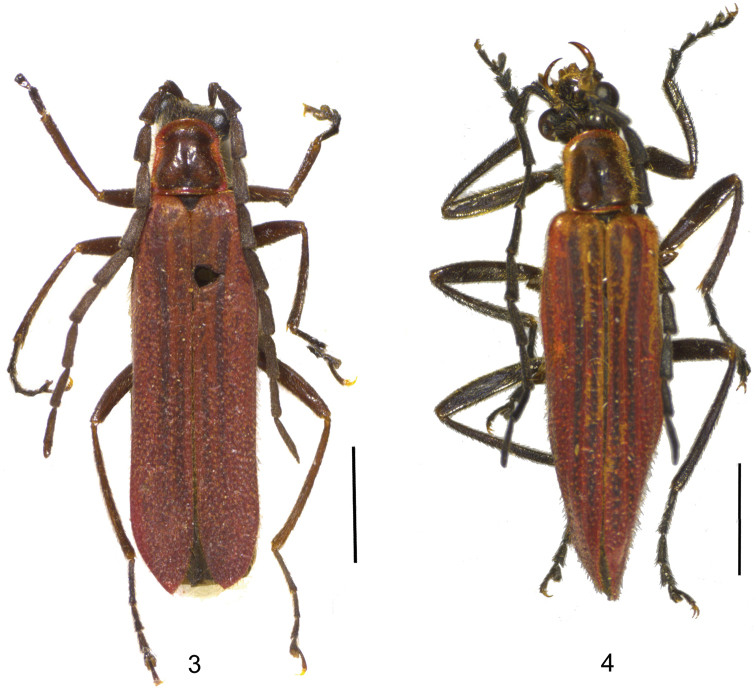
Male habitus, dorsal view: **3** *Lycocerus sichuanus* sp. n. **4** *Lycocerus hubeiensis* sp. n. Scale bars: 2.0 mm.

Male (Fig. 3). Body black, mandibles dark brown, pronotum red, with a large dark brown marking, which almost extending to all margins of disc, elytra red, nearly black at the whole length of the first and second elytral interstices, more or less darkened at the third and fourth elytral interstices.

Head evenly narrowed behind eyes, surface densely and finely punctate, semilustrous, covered with dense, fine, yellowish brown decumbent pubescence; eyes strongly protruding, head width across eyes distinctly wider than anterior margin of pronotum; terminal maxillary palpomere long-triangular, nearly truncate and sharp at apical one-third length of inner margin; antennae (Fig. 10) extending to apical one-third length of elytra, antennomeres II nearly as long as wide at apices, III–XI flattened, III–VIII obliquely widened apically, nearly long-triangular, III about twice as long as wide at apices, IV slightly longer than III, IX–XI nearly parallel-sided, XI slightly longer than X and pointed at apices, IV–XI each with an oval to round smooth impression at apical part of outer margin.

Pronotum distinctly longer than wide, widest near posterior margin, anterior margin rounded, anterior angles rounded, lateral margins moderately diverging posteriorly, slightly sinuate at anterior portion, posterior angles rectangular, posterior margin slightly arcuate and narrowly bordered, disc moderately convex at posterolateral parts, surface punctate and pubescent like that of head, semilustrous.

Elytra about 4.7 times longer than pronotum, 3.7 times longer than humeral width, which about one-third wider than posterior margin of pronotum, lateral margins nearly parallel, elytral venations moderately developed, slightly costate.

Pro- and meso-outer tarsal claws each with a basal projection.

Abdominal sternite IX long-triangular. Aedeagus (Figs 22–24): ventral process of each paramere normal and rounded at apex, even and nearly straight in lateral view; dorsal fig slightly shorter than ventral process, evenly narrowed at inner apical portion and slightly widened at inner angle, with inner angle acute-angled, outer angle obtuse-angled, inner margin bisinuate, apical margin rounded; laterophyse with apex pointed laterodorsally to outer angle of dorsal fig.

Female. Similar to the male, but eyes less protruding; antennae (Fig. 9) shorter, extending to basal one-third length of elytra, antennomeres III about twice as long as wide at apices, IV–XI without impressions; pronotum nearly as long as wide, disc slightly convex on posterolateral parts; elytra with lateral margins slightly diverging posteriorly; abdominal sternite VIII (Fig. 35) roundly emarginated in middle and triangularly emarginated on both sides of posterior margin, the portion between middle and lateral emarginations obtuse-angled at apex; internal reproductive organ of genitalia see Fig. 39.

Body length (both sexes): 8.0–10.0 mm; width: 1.8–2.0 mm.

###### Etymology.

This specific name is derived from its locality, Sichuan Province, China.

###### Diagnosis.

This species is similar to *Lycocerus hubeiensis* sp. n., but differs from the latter by the antennae of female much narrower, antennomeres III about twice as long as wide at apices; aedeagus: ventral process of each paramere even and nearly straight in lateral view; abdominal sternite VIII of female with the portion between middle and lateral emarginations of posterior margin obtuse-angled at apex.

##### Lycocerus hubeiensis

Taxon classificationAnimaliaColeopteraCantharidae

Y. Yang & X. Yang sp. n.

http://zoobank.org/1DB00DF3-2E20-45D5-B126-BA23BD1E80AB

Figs 4 , 11 –12 , 25 –27 , 36 , 40

###### Type material.

Holotype ♂ (MHBU): CHINA, Hubei, Dalaoling Nat. Res., 1200m, 9.VII.2011, leg. Xue-Song Guan. Paratypes: CHINA, Hubei: 1♀ (MHBU): Dalaoling Nat. Res., 1200m, 9.VII.2011, leg. Xiao-Long Yang; 1♀ (MHBU): same data, 10.VII.2011; 1♂ (MHBU): Badong, Lvcongpo, 1700m, 18.VII.2006, leg. Jun-Hua Wan; 1♀ (MHBU): Badong, Tiansanping, 1500m, 14.VII.2006, leg. Ping Hu; 1♀ (MHBU): Shennongjia, Bajiaomiao, 900–1300m, 17.VII.2003, leg. Yuan He; 1♀ (MHBU): same locality, 19.VII.2003, leg. Jun Ma; 1♂ (MHBU): Shennongjia, Wenshui Forestry, 1700–2000m, 20.VII.2003, leg. Hua He; 1♀ (MHBU): Wufeng, Houhe, 21.VII.2002, leg. Ying Shi; 1♀ (IZAS): Xingshan, Longmenhe, 1300m, 15.VI.1993, leg. Jian Yao; 1♀ (IZAS): same locality, 1350m, 18.VII.1993, leg. Xiao-Lin Chen; 1♀ (IZAS): same locality, 1400m, 22.VII.1993, leg. Shi-Mei Song; 1♀ (IZAS): same locality, 1670m, 23.VII.1993, leg. Xing-Ke Yang. [All are transliterated from Chinese labels].

###### Distribution.

China (Hubei).

###### Description.

Male (Fig. 4). Body black, mandibles dark brown, pronotum red, with a large dark brown marking, which almost extending to all margins of disc, elytra red, nearly black at the whole length of the first and second elytral interstices, more or less darkened at the third and fourth elytral interstices.

Head evenly narrowed behind eyes, surface densely and finely punctate, semilustrous, covered with dense, fine, yellowish brown decumbent pubescence; eyes strongly protruding, head width across eyes distinctly wider than anterior margin of pronotum; terminal maxillary palpomere long-triangular, nearly truncate and sharp at apical one-third length of inner margin; antennae (Fig. 12) extending to apical one-third length of elytra, antennomeres II nearly as long as wide at apices, III–XI flattened, III–IX obliquely widened apically, nearly long-triangular, III about twice as long as wide at apices, IV slightly longer than III, X–XI nearly parallel-sided, XI slightly longer than X and pointed at apices, IV–XI each with an oval to round smooth impression at apical part of outer margin.

Pronotum distinctly longer than wide, widest near posterior margin, anterior margin rounded, anterior angles rounded, lateral margins moderately diverging posteriorly, slightly sinuate at anterior portion, posterior angles rectangular, posterior margin slightly arcuate and narrowly bordered, disc moderately convex at posterolateral parts, surface punctate and pubescent like that of head, semilustrous.

Elytra about 5.3 times longer than pronotum, 3.7 times longer than humeral width, which about one-third wider than posterior margin of pronotum, lateral margins nearly parallel, elytral venations moderately developed, slightly costate.

Pro- and meso-outer tarsal claws each with a basal projection.

Abdominal sternite IX long-triangular. Aedeagus (Figs 25–27): ventral process of each paramere normal and rounded at apex, distinctly narrowed at base and slightly bent dorsally in lateral view; dorsal fig distinctly shorter than ventral process, evenly narrowed at inner apical portion and slightly widened at inner angle, with inner angle acute-angled, outer angle obtuse-angled, inner margin bisinuate, apical margin rounded; laterophyse with apex pointed laterodorsally to outer angle of dorsal fig.

Female. Similar to male, but eyes less protruding; antennae (Fig. 11) shorter, extending to elytral midlength, antennomeres III–XI distinctly widened, III about 1.6 times longer than wide at apices, IV–XI without impressions; pronotum nearly as long as wide, disc slightly convex on posterolateral parts; elytra with lateral margins slightly diverging posteriorly; abdominal sternite VIII (Fig. 36) largely and roundly emarginated in middle and both sides of posterior margin, the portion between middle and lateral emarginations rounded at apex; internal reproductive organ of genitalia see Fig. 40.

Body length (both sexes): 7.5–11.0 mm; width: 1.5–2.3 mm.

###### Etymology.

This specific name is derived from its locality, Hubei Province, China.

###### Diagnosis.

This species is similar to *Lycocerus sichuanus* sp. n. in the aedeagus, but differs from the latter by the antennae of female much wider, antennomeres III about 1.6 times longer than wide at apices; aedeagus: ventral process of each paramere narrowed at base and slightly bent dorsally in lateral view; abdominal sternite VIII of female with the portion between middle and lateral emarginations of posterior margin rounded at apex.

##### Lycocerus napolovi

Taxon classificationAnimaliaColeopteraCantharidae

Y. Yang & X. Yang sp. n.

http://zoobank.org/18BFD8BA-1595-4EBF-9E4A-A6F75C805965

**Figures 5–6.**
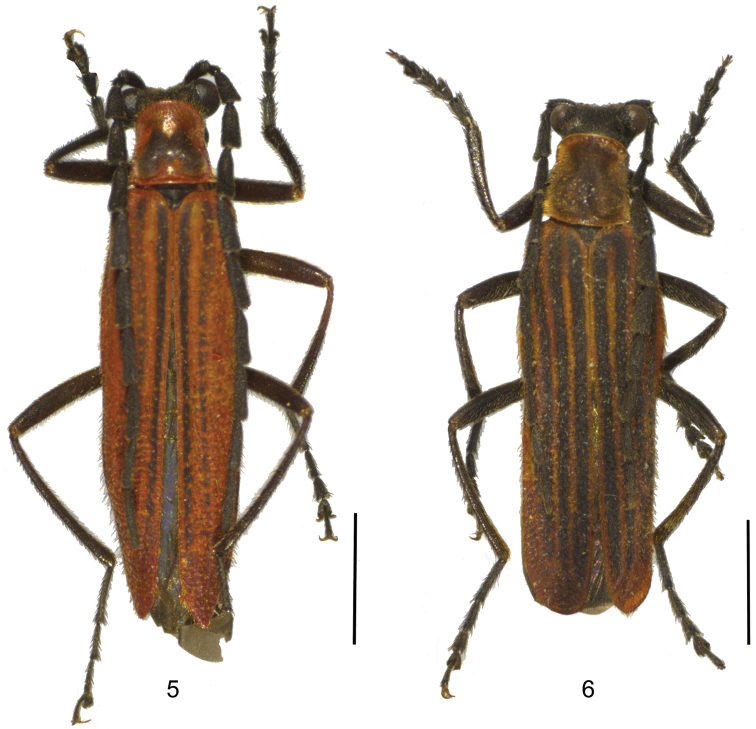
Male habitus, dorsal view: **5** *Lycocerus napolovi* sp. n. **6** *Lycocerus quadrilineatus* sp. n. Scale bars: 2.0 mm.

Figs 5 , 13 , 28 –30

###### Type material.

Holotype ♂ (ZIN): “Vietnam N, (Sa Pa), Lao Cai prov., 250km from Hanoi bearing 310#, Sa Pa vill. env., Hoang Lien Son Nat. Res., 1250-1300m, 15.-21.6.1998, leg. A. Napolov”.

###### Distribution.

Vietnam (Sa Pa).

###### Description.

Male (Fig. 5). Body black, mandibles dark brown, pronotum red, with a large black marking in center of disc, elytra red, nearly black at the whole length of the first and second elytral interstices.

Head evenly narrowed behind eyes, surface densely and finely punctate, semilustrous, covered with dense, fine, yellowish brown decumbent pubescence; eyes strongly protruding, head width across eyes distinctly wider than anterior margin of pronotum; terminal maxillary palpomere long-triangular, nearly truncate and sharp at apical one-third length of inner margin; antennae (Fig. 13) almost extending to apical one-fourth length of elytra, antennomeres II nearly as long as wide at apices, III–XI flattened, III–IX obliquely widened apically, nearly long-triangular, III about twice as long as wide at apices, IV slightly longer than III, X–XI nearly parallel-sided, XI slightly longer than X and pointed at apices, IV–XI each with a short narrow longitudinal smooth impression at apical part of outer margin.

**Figures 7–15.**
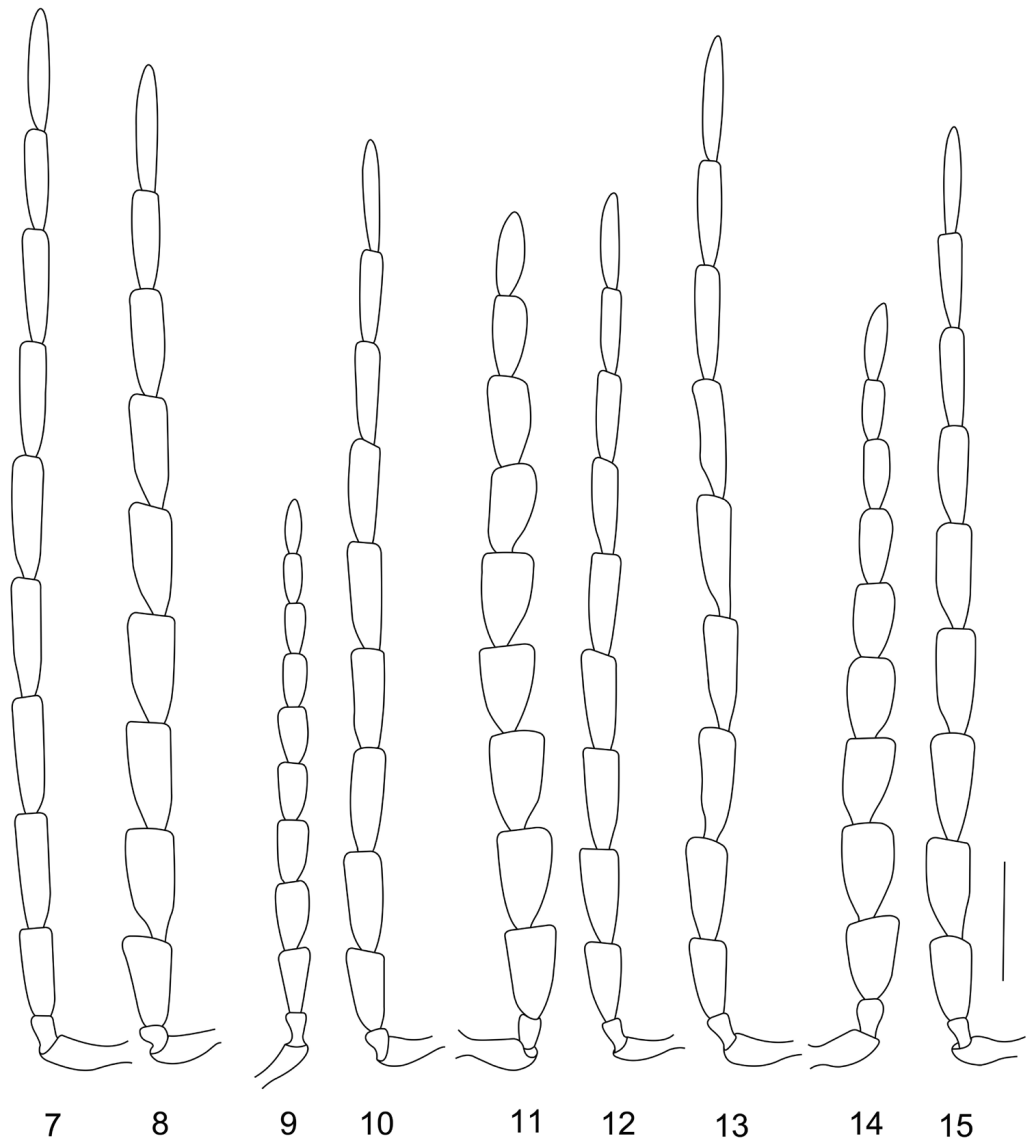
Antennae, dorsal views (**7, 8, 10, 12, 13, 15** male; **9, 11, 14** female): **7** *Lycocerus gracilicornis* sp. n. **8** *Lycocerus longihirtus* sp. n. **9–10** *Lycocerus sichuanus* sp. n.; **11–12** *Lycocerus hubeiensis* sp. n. **13** *Lycocerus napolovi* sp. n. **14–15** *Lycocerus quadrilineatus* sp. n. Scale bar: 1.0 mm.

**Figures 16–24.**
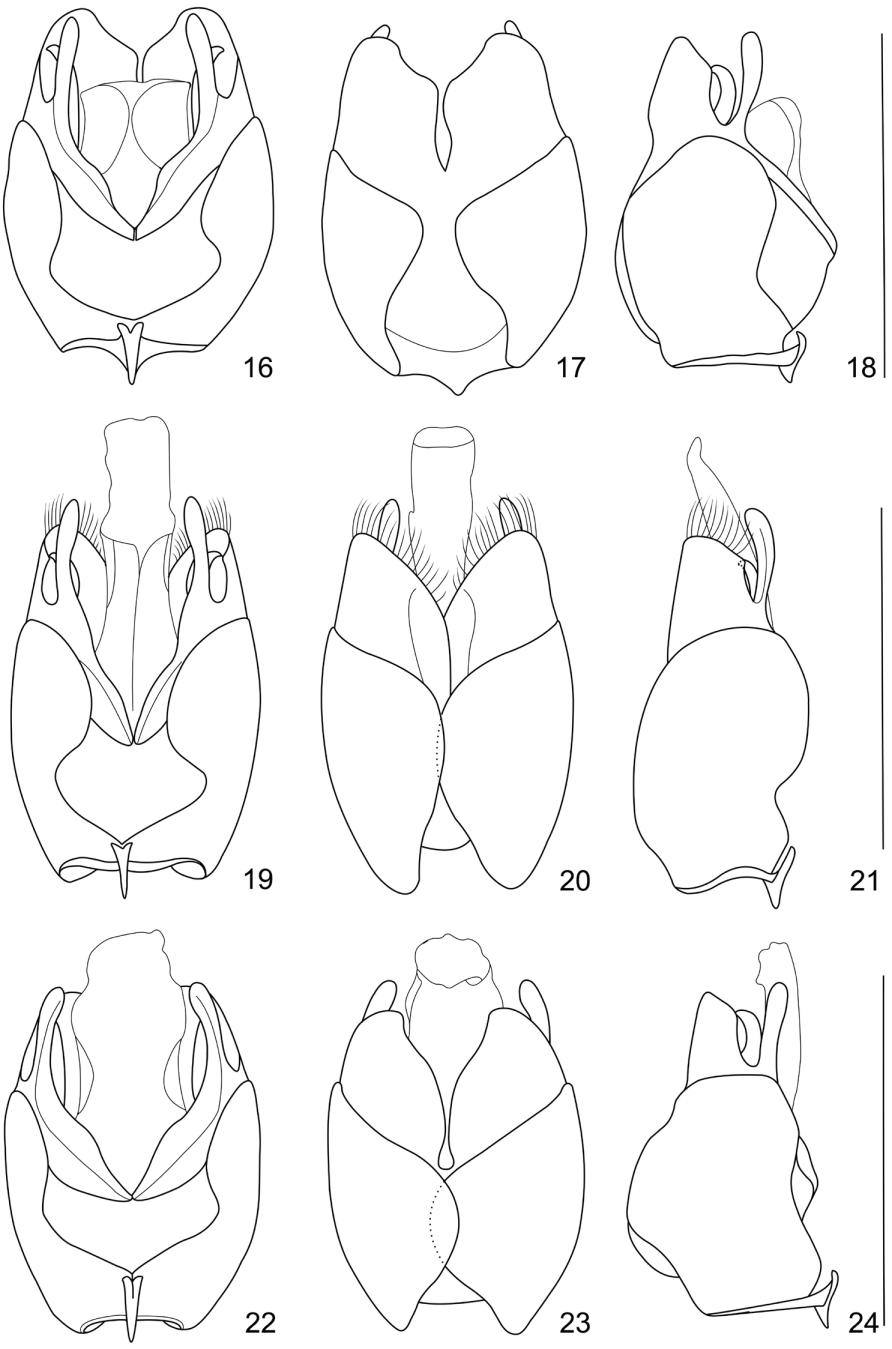
Aedeagus (**16, 19, 22** ventral view; **17, 20, 23** dorsal view **18, 21, 24** lateral view): **16–18** *Lycocerus gracilicornis* sp. n. **19–21** *Lycocerus longihirtus* sp. n. **22–24** *Lycocerus sichuanus* sp. n. Scale bars: 1.0 mm.

Pronotum distinctly longer than wide, widest near posterior margin, anterior margin rounded, anterior angles rounded, lateral margins moderately diverging posteriorly, slightly sinuate at anterior portion, posterior angles rectangular, posterior margin slightly arcuate and narrowly bordered, disc moderately convex at posterolateral parts, surface punctate and pubescent like that of head, semilustrous.

Elytra about 5.3 times longer than pronotum, 4.0 times longer than humeral width, which about one-third wider than posterior margin of pronotum, lateral margins nearly parallel, elytral venations moderately developed, slightly costate.

All tarsal claws simple.

Abdominal sternite IX long-triangular. Aedeagus (Figs 28–30): ventral process of each paramere flattened, twist in middle and tapered at apex in ventral view, slightly narrowed at base and distinctly bent dorsally in lateral view; dorsal fig slightly shorter than ventral process, abruptly narrowed at inner apical portion, with a longitudinal ridge in middle of basal portion, membranous between inner margin and the ridge, inner angle rectangular, outer angle acute-angled and bent ventrally, inner margin distinctly protuberant in middle, apical margin nearly straight, around with long pubescence; laterophyse with apex pointed laterally to outer apical angle of dorsal fig.

**Figures 25–33.**
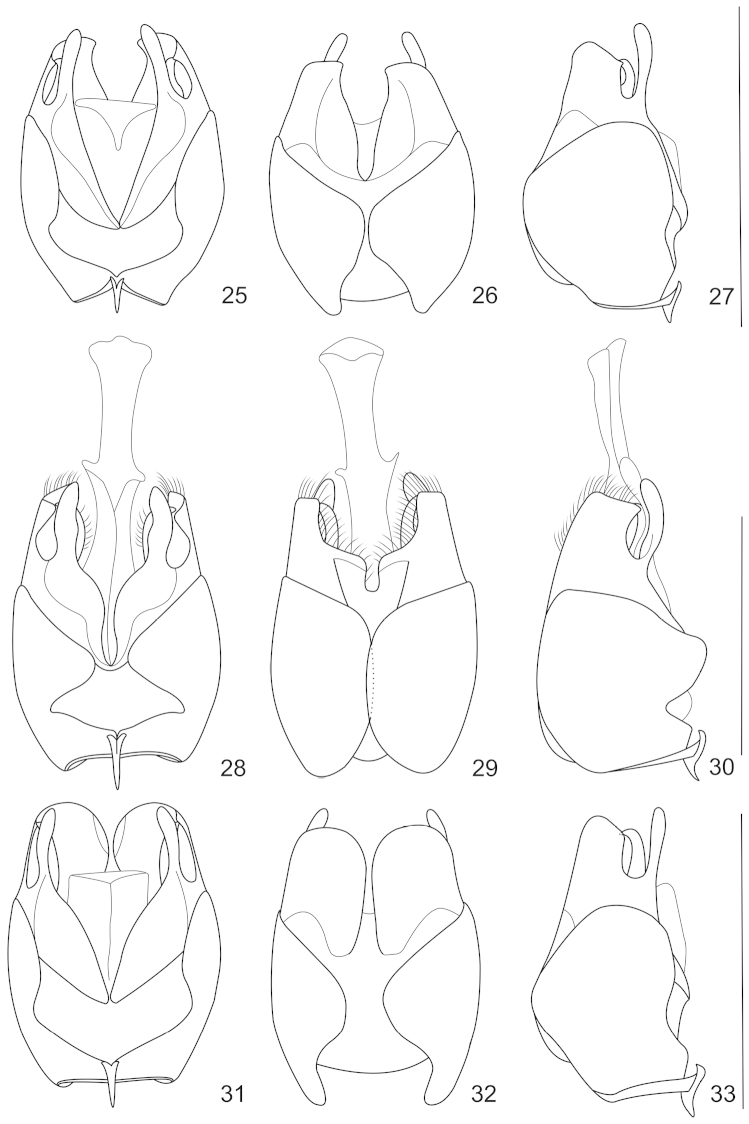
Aedeagus (**25, 28, 31** ventral view **26, 29, 32** dorsal view **27, 30, 33** lateral view): **25–27** *Lycocerus hubeiensis* sp. n. **28–30** *Lycocerus napolovi* sp. n.; **31–33** *Lycocerus quadrilineatus* sp. n. Scale bars: 1.0 mm.

Female. Unknown.

Body length (male): 9.0 mm; width: 1.5 mm.

###### Etymology.

Patronymic, dedicated to its collector, Alexander Napolov (Riga, Latvia).

###### Diagnosis.

This species could be easily distinguished from others by its aedeagus: ventral process of each paramere slightly flattened and twist in middle in ventral view, distinctly bent dorsally in lateral view, dorsal fig with a longitudinal ridge in middle of basal portion, membranous between inner margin and the ridge.

###### Remarks.

The left mesoleg and right metatarsomeres II–V of the holotype are missing.

##### Lycocerus quadrilineatus

Taxon classificationAnimaliaColeopteraCantharidae

Y. Yang & X. Yang sp. n.

http://zoobank.org/EC8B55E5-A8DD-482B-8E79-BFE872697E21

Figs 6 , 14 –15 , 31 –33 , 37 , 41

###### Type material.

Holotype ♂ (ZIN): “N. Vietnam, Sa Pa env., 1200m, 20.V.1999, leg. Ozlov”. Paratypes: 1♀ (ZIN): same data as holotype; 1♀ (ZIN): “ВЬЕТНАМ [Vietnam] горы, у ША-ПА [Sa Pa], 1600–2000m, 5.6.1963г, Каьакое [Kabakov]”.

###### Distribution.

Vietnam (Sa Pa).

###### Description.

Male (Fig. 6). Body black, mandibles dark brown, pronotum red, with a large dark brown marking, which almost extending to all margins of disc, elytra red, nearly black at the whole length of all elytral interstices.

Head evenly narrowed behind eyes, surface densely and finely punctate, semilustrous, covered with dense, fine, yellowish brown decumbent pubescence; eyes strongly protruding, head width across eyes distinctly wider than anterior margin of pronotum; terminal maxillary palpomere long-triangular, nearly truncate and sharp at apical one-third length of inner margin; antennae (Fig. 15) extending to apical one-fourth length of elytra, antennomeres II nearly as long as wide at apices, III–XI flattened, III–IX obliquely widened apically, nearly long-triangular, III about twice as long as wide at apices, IV slightly longer than III, X–XI nearly parallel-sided, XI slightly longer than X and pointed at apices, IV–XI each with a round smooth impression at apical part of outer margin.

Pronotum slightly longer than wide, widest near posterior margin, anterior margin rounded, anterior angles rounded, lateral margins moderately diverging posteriorly, slightly sinuate at anterior portion, posterior angles rectangular, posterior margin slightly arcuate and narrowly bordered, disc moderately convex at posterolateral parts, surface punctate and pubescent like that of head, matt.

Elytra about 4.3 times longer than pronotum, 3.5 times longer than humeral width, which about one-third wider than posterior margin of pronotum, lateral margins nearly parallel, elytral venations well-developed, moderately costate.

All tarsal claws simple.

Abdominal sternite IX long-triangular. Aedeagus (Figs 31–33): ventral process of each paramere normal and rounded at apex, even and nearly straight in lateral view; dorsal fig slightly shorter than ventral process, not narrowed apically, with inner angle rounded, outer angle obtuse-angled, inner margin nearly straight, apical margin rounded; laterophyse with apex pointed laterodorsally to outer angle of dorsal fig.

Female. Similar to the male, but eyes less protruding; antennae (Fig. 14) shorter, extending to basal one-third length of elytra, antennomeres III about 1.1 times longer than wide at apices, IV–XI without impressions; pronotum nearly as long as wide, disc slightly convex on posterolateral parts; elytra with lateral margins slightly diverging posteriorly; pro- and meso-outer and inner tarsal claws each with a basal projection; abdominal sternite VIII (Fig. 37) very slightly emarginated in middle and largely emarginated on both sides of posterior margin, the portion between middle and lateral emarginations indistinctly angled at apex; internal reproductive organ of genitalia see Fig. 41.

Body length (both sexes): 8.5–10.0 mm; width: 2.0–2.2 mm.

**Figures 34–38.**
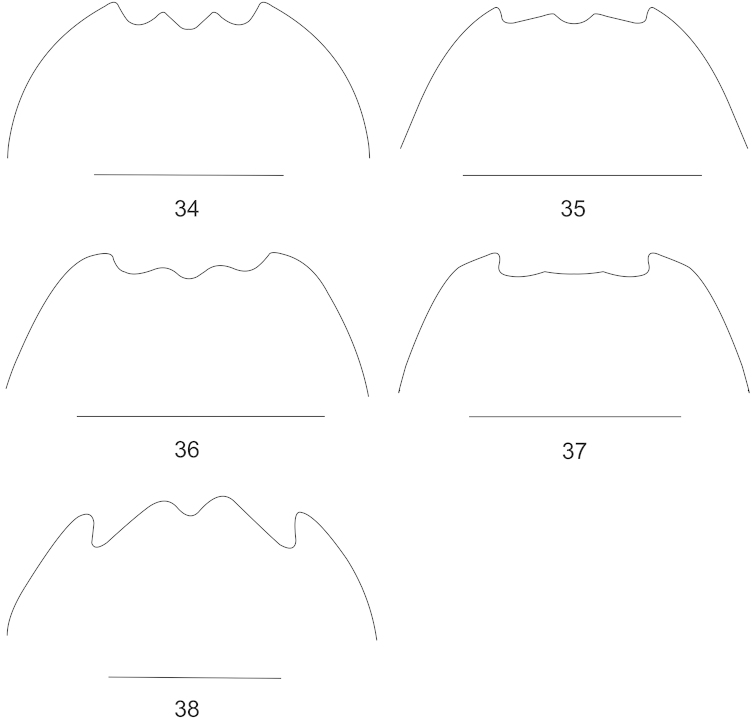
Abdominal sternite VIII of female, ventral view: **34** *Lycocerus hickeri* (Pic, 1934) **35** *Lycocerus sichuanus* sp. n. **36** *Lycocerus hubeiensis* sp. n. **37** *Lycocerus quadrilineatus* sp. n. **38** *Lycocerus obscurus* Pic, 1916. Scale bars: 1.0 mm.

**Figures 39–41.**
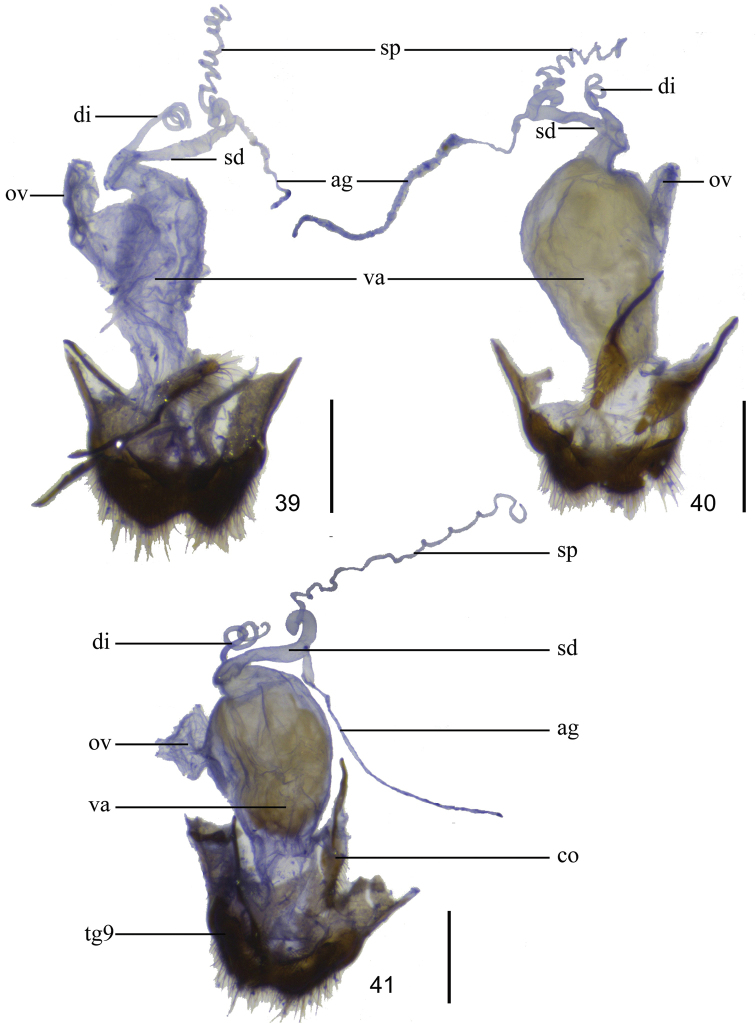
Female genitalia, lateral view: 39. *Lycocerus sichuanus* sp. n.; **40** *Lycocerus hubeiensis* sp. n. **41** *Lycocerus quadrilineatus* sp. n. Scale bars: 0.5 mm.

###### Etymology.

The specific name is derived from Latin *quadrus* (four) and *linea* (stripe), referring to its elytra darkened at all of the four elytral interstices.

###### Diagnosis.

This species can be easily distinguished from other species by the elytra darkened at all elytral interstices, elytral venation well-developed; all claws simple in male, pro- and meso-outer and inner tarsal claws each with a basal projection in female; aedeagus: dorsal fig of each paramere not narrowed apically.

#### New faunistic records

##### Lycocerus bicoloripennis

Taxon classificationAnimaliaColeopteraCantharidae

(Pic, 1924)

Cantharis bicoloripennis Pic, 1924: 478.
Athemus (s. str.) bicoloripennis : Wittmer 1995: 273, Figs 137, 138.

###### Type material examined.

Holotype: 1 ♂ (MNHN): [p]“MUSEUM PARIS \ SIKKIM \ DARDJILING \ HARMAND 1890”, [h]“Det. M. Pic (Digoin) \ Cantharis \ bicoloripennis \ n. sp.”, [h]“Athemus \ bicoloripennis \ (Pic) \ det. W. Wittmer”, [p]“HOLOTYPUS”.

###### Additional material examined.

CHINA, Xizang: 1 ♂ (IZAS): Nyalam, Zham, 2200m, 28.VI.1975, leg. Zi-Qing Wang; 1 ♂, 1 ♀ (HBUM): Zham, 5.–6.VII.2004, leg. Yi-Bin Ba & Ai-Min Shi.

###### Distribution.

China (new record: Xizang); Nepal, India (Kazantsev and Brancucci 2007).

##### Lycocerus caliginostus

Taxon classificationAnimaliaColeopteraCantharidae

Gorham, 1889

Lycocerus caliginostus Gorham, 1889: 110.
Lycocerus vittaticollis Champion, 1926: 255. [Synonymized by Kazantsev 1999: 130].

###### Type material examined.

Lectotype of *Lycocerus caliginostus*: 1♀ (MNHN): without locality data, [h]“Lycocerus \ caliginosus \ Gorh.”, [p] “TYPE”.

Paratype of *Lycocerus vittaticollis*: 1♀ (NHMB): [p]“Birmah \ Ruby-mines”, [p]“Doherty”, [p]“Frey Coll. \ 1905.100.”, [p]“♀”, [p]“Para \ type”, [p]“Lycoerus \ (?) vittaticollis, \ Champ. ”, [p]“E.M.M. 1926 \ det. G.C.C.”, [h]“Lycocerus \ vittaticollis \ Champ. \ det. W. Wittmer”, [h]“Athemus \ caliginosus (Gorh.) \det. S. Kazantsev 1996”, [p]“CANTHARIDAE \ CANTH00002784”, [p]“Naturhistorisches \ Museum Basel \ Coll. W. Wittmer”.

###### Additional material examined.

CHINA, Yunnan: 1♀ (IZAS): Menghai, Nannuoshan, 1100–1500m, 27.IV.1957, leg. Guang-Ji Hong; 1♀ (IZAS): Xiaomengyang, 850m, 3.V.1957, leg. Panfilov; 1♂ (IZAS): same data, 2.IV.1957, leg. Ling-Chao Zang; 1♂ (IZAS): Menghai, Nannuoshan, 1250m, 24.IV.1957, leg. Panfilov; 1♂ (IZAS): Xishuangbanna, Mengsong, 1600m, 23.IV.1958, leg. Fu-Ji Pu; 1♀ (IZAS): same data, 25.IV.1958, leg. Chun-Pei Hong; 1♀ (IZAS): same data, leg. Yi-Ran Zhang; 1♀ (IZAS): same data, 22.IV.1958, leg. Xu-Wu Meng; 1♀ (IZAS): same data, 23.IV.1958, leg. Shu-Yong Wang; 1♂ (IZAS): same data, 22.IV.1958, leg. Shu-Yong Wang; 1♂ (IZAS): Cheli, Shihuiyao, 750m, 27.IV.1958, leg. Panfilov; 1♀ (IZAS): Simao, 1300m, 31.IV.1957, leg. Bushchik.

###### Distribution.

China (new record: Yunnan); Laos, Thailand, Myanmar (Kazantsev 1999).

##### Lycocerus jendeki

Taxon classificationAnimaliaColeopteraCantharidae

Švihla, 2005

Lycocerus jendeki Švihla, 2005: 94, figs 48–51.

###### Type material examined.

Holotype: 1♂ (NMPC): [p]“Laos north, 5–11.V.1997, \ 20 km NW Louang Namtha, \ N21°09.2, E 101°18.7, \ alt. 900±100 m, \ E. Jendek & O. Šauša leg.”, [p]“HOLOTYPUS \ Lycocerus \ jendeki sp. nov. \ V. Švihla det. 2005”.

###### Additional material examined.

CHINA, Yunnan: 1♂ (IZAS): Simao, Rd. Kunluo, 1350m, 11.V.1957, leg. Fu-Ji Pu; 1♀ (IZAS): Xishuangbanna, Meng’a, 1050–1080m, 10.V.1958, leg. Shu-Yong Wang.

###### Distribution.

China (new record: Yunnan); Laos.

##### Lycocerus malaisei

Taxon classificationAnimaliaColeopteraCantharidae

(Wittmer, 1995)

Athemus (s. str.) malaisei Wittmer, 1995: 277, figs 144–145, 195.

###### Type material examined.

Holotype: 1♂ (NHMB): [p]“N. E. Burma \ Kambaiti, 7000 ft \ 24/5 1934 \ R. Malaise”, [h]“malaisei”, [p]“HOLOTYPE”, [p]“Naturhistorisches \ Museum Basel \ coll. W. Wittmer”.

###### Additional material examined.

CHINA, Yunnan: 1♂ (IZAS): 60km E tengchong, 2200m, 19.–22.V2006, leg. S. Murzin & I. Shokhin; 1♂ (CAS): Longling County, Longjiang Township, Xiaoheishan Forest Reserve, Guchengshan, 2020m, N24.82888°/E098.76001°, 26.V.2005, stop# 2005-030A, D.H.Kavanaugh & C.E. Griswold collectors [CASENT 6004806]; 1♂ (CAS): same data [CASENT 1035946]; 1♂ (CAS): same data [CASENT 1035947]; 1♀ (CAS): same data [CASENT 1035948]; 1♀ (CAS): Longyang County, Bawan Township, Nankang Yakou just N of pass, N24.83178°/E098.76472°, 2180m, 25.V.2005, stop# 2005-029B, D.H. Kavanaugh & C.E. Griswold collectors [CASENT 1035849].

###### Distribution.

China (new record: Yunnan); Myanmar.

##### Lycocerus obscurus

Taxon classificationAnimaliaColeopteraCantharidae

Pic, 1916

Fig. 38

Lycocerus obscurus Pic, 1916: 13.
Lycocerus obscurus var. diversus Pic, 1916: 13. [Synonymized by Kazantsev 1999: 134].
Lycocerus rubroniger Švihla, 2011: 12, Figs 13, 64–66. **syn. n.**

###### Type material examined.

Lectotype of *Lycocerus obscurus*: 1♀ (MNHN): [h] “Xieng Khuang” [LAOS, Xieng Khouang]. Paralectotypes: 2♀♀(MNHN): same data as lectotype; 1♀ (MNHN): “X. K.”; 1♀ (MNHN): “Xieng Khuang \ 17-III-1919 \ R. Vitalis de Salvaza”.

Lectotype of Lycocerus obscurus var. diversus: 1♀ (MNHN): [h]“Xieng Khuang \ ex Vitalis”, [h]“Lycocerus obscurus v. \ diversus Pic ”, [h]“type”, [h]“obscurus \ v. diversus”, [p]“TYPE”, [h]“LECTOTYPUS \ Lycocerus obscurus v. \ diversus Pic \ S. Kazantsev det.”.

###### Additional material examined.

CHINA, Guangxi: 2♂♂, 2♀♀ (IZAS): Jingxi, 840m, 1.IV.1998, leg. Chun-Sheng Wu.

###### Supplementary description.

Female. Similar to male, but eyes less protruding; antennae shorter, extending to basal one-third length of elytra; pronotum wider; abdominal sternite VIII (Fig. 38) largely emarginated in middle and on both sides of posterior margin, the portion between middle and lateral emarginations distinctly protuberant and rounded at apex.

Body length (both sexes): 10.0–12.5 mm; width: 2.5–3.0 mm.

###### Distribution.

China (new record: Guangxi); Laos (Kazantsev 1999).

###### Remarks.

Based on the examination of the types of *Lycocerus obscurus* Pic, 1916 and some additional material at our disposal, we found no difference between it and *Lycocerus rubroniger* Švihla, 2011, which was presented with an adequate photo of the habitus and illustrations of aedeagus in the original publication. Therefore, *Lycocerus rubroniger* is synonymized with *Lycocerus obscurus* here, and provided with the supplementary description and illustration of abdominal sternite VIII of female.

##### Lycocerus olivaceus

Taxon classificationAnimaliaColeopteraCantharidae

(Wittmer, 1995)

Athemus (s. str.) olivaceus Wittmer, 1995: 219, Fig. 57.

###### Type material examined.

Holotype: 1♂ (NHMB): [h]“Shillong \ Assam”, [h]“26.4.1971 \ T. Sen Gupta”, [h]“Athemus s.str. \ olivaceus \ Wittm. \ det. W. Wittmer”, [p]“HOLOTYPUS”, [p]“Naturhistorisches \ Museum Basel \ coll. W. Wittmer”, [p]“CANTHARIDAE \ CANTH00001639”.

###### Additional material examined.

CHINA, Yunnan: 1♂, 2♀♀ (IZAS): Gongshan, Dulong, Kongdang, 21.V.2007, leg. Yan-Lei Li.

###### Distribution.

China (new record: Yunnan); India.

##### Lycocerus purpureus

Taxon classificationAnimaliaColeopteraCantharidae

Kazantsev, 2007

Lycocerus purpureus Kazantsev, 2007: 54 [replacement name for Athemus (Andrathemus) purpurascens Wittmer, 1978, nec Pic, 1911].
Athemus (Andrathemus) purpurascens Wittmer, 1978: 155, Fig. 5. [Preoccupied by *Cantharis purpurascens* Pic, 1911: 143, synonymized with *Lycocerus rubripennis* (Hope, 1831)].

###### Type material examined.

Holotype: 1♂ (NHMB): [p]“Dechhi Paka 3300m \ 19.-20.5.” [p]“Nat.-Hist. Museum \ Basel-Bhutan \ Expedition 1972”, [h]“Athemus subg. \ Andrathemus \ purpurascens \ Wittm. \ det. W. Wittmer”, [p]“HOLOTYPUS”, [p]“CANTHARIDAE \ CANTH00001271”.

###### Additional material examined.

CHINA, Xizang: 1♂, 1♀ (IZAS): Nyingtri, 3050m, 3.VIII.1983, leg. Yin-Heng Han.

###### Distribution.

China (new record: Xizang); Bhutan (Kazantsev and Brancucci 2007).

##### Lycocerus ruficornis

Taxon classificationAnimaliaColeopteraCantharidae

(Wittmer, 1995)

Athemus (s. str.) ruficornis Wittmer, 1995: 232, Figs 77, 180.

###### Type material examined.

Holotype: 1♂ (NHMB): [p]“THAI, 10.-16.V.1991 \ Chiang Dao 600m \ 15°24’N 98°55’E \ Vit Kubáň leg.”, [p]“Thailand 91 \ Thanon Thong Chai \ D. Král & V. Kubáň”, [h]“Athemus \ ruficornis \ Wittm. \ det. W. Wittmer”, [p]“HOLOTYPUS”, [p]“CANTHARIDAE \ CANTH00002524”.

###### Additional material examined.

CHINA, Yunnan: 1♂ (IZAS): Xishuangbanna, Meng’a, 1050–1080m, 2.V.1958, leg. Shu-Yong Wang; 1♀ (IZAS): Xishuangbanna, Xiaomengyang, 850m, 8.VII.1957, leg. Ling-Chao Zang. [MYANMAR]: 2♂♂ (MCSNG): Burma, Tenasserim, Thagalà, IV.1887, Fea.

###### Distribution.

China (new record: Yunnan); Myanmar (new record); Thailand.

##### Lycocerus semiextensus

Taxon classificationAnimaliaColeopteraCantharidae

(Wittmer, 1995)

Athemus (Andrathemus) rubripennis Pic: Wittmer 1978: 158, Fig. 9 (parte).
Athemus (Andrathemus) semiextensus Wittmer, 1995: 264, Figs 123–124.

###### Type material examined.

Holotype: 1♂ (NHMB): [p]“E-Nepal \ Koshi \ M. Brancucci”, [p]“Gufa-Gorza \ 2800-2100m \ 4.VI.1985”, [h]“REM \ 94 \ 4.7”, [h]“Athemus s.str. \ semiextensus \ Wittm. \ det. W. Wittmer”, [p]“HOLOTYPUS”, [p]“CANTHARIDAE \ CANTH00001301”.

###### Additional material examined.

CHINA, Xizang: 1♂ (IZAS): Droma, 2800m, 8.VI.1961, leg. Lin-Yao Wang; 1 ♀ (IZAS): same data, 7.VI.1961; 1 ♂ (IZAS): same data, 5.VI.1961; 1 ♀ (IZAS): same data, 6.VI.1961.

###### Distribution.

China (new record: Xizang); India, Bhutan, Nepal (Kazantsev and Brancucci 2007).

## Supplementary Material

XML Treatment for Lycocerus hickeri

XML Treatment for Lycocerus gracilicornis

XML Treatment for Lycocerus longihirtus

XML Treatment for Lycocerus sichuanus

XML Treatment for Lycocerus hubeiensis

XML Treatment for Lycocerus napolovi

XML Treatment for Lycocerus quadrilineatus

XML Treatment for Lycocerus bicoloripennis

XML Treatment for Lycocerus caliginostus

XML Treatment for Lycocerus jendeki

XML Treatment for Lycocerus malaisei

XML Treatment for Lycocerus obscurus

XML Treatment for Lycocerus olivaceus

XML Treatment for Lycocerus purpureus

XML Treatment for Lycocerus ruficornis

XML Treatment for Lycocerus semiextensus
